# Comparison of clinical characteristics of COVID-19 between elderly patients and young patients: a study based on a 28-day follow-up

**DOI:** 10.18632/aging.104077

**Published:** 2020-10-26

**Authors:** Lin Zhang, Tao Fan, Shuo Yang, Haojie Feng, Bo Hao, Zilong Lu, Rui Xiong, Xiaokang Shen, Wenyang Jiang, Wei Wang, Qing Geng

**Affiliations:** 1 Department of Thoracic Surgery, Renmin Hospital of Wuhan University, Wuhan 430060, China; 2 Department of Cardiology, Renmin Hospital of Wuhan University, Cardiovascular Research Institute of Wuhan University, Wuhan 430060, China

**Keywords:** coronavirus infections, COVID-19, clinical characteristics, prognostic factors, elderly patients

## Abstract

The number of corona virus disease 2019 cases is increasing rapidly. However, the comparison of clinical characteristics between patients ≥ 70 and those < 70 has not been implemented yet. To achieve that, we collected clinical data of consecutive 222 patients in Renmin Hospital of Wuhan University diagnosed between January 13, 2020 and February 4, 2020. We divided them into an under-70 group and an over-70 group according to their ages, comparing their clinical characteristics. Meanwhile, univariate and multivariate Cox regression analyses were performed to identify the prognostic factors. Among the patients enrolled, 37 (16.67%) were 70 or older and 185 (83.33%) were younger than 70. Higher proportions of dyspnoea, expectoration, chronic cardiovascular disease, diabetes, organ complications, severe-to-critical cases, a higher death rate, a longer hospital stay and decreased immune status were observed in the over-70 group patients compared with their younger counterparts. The risk factors for death included dyspnoea, muscle ache, elevated myocardial enzymes, elevated C3 in over-70 patients and dyspnoea, pharyngalgia, chronic cardiac disease, increased C-reactive protein, IgA, decreased platelets in under-70 patients. Overall, our research compared the clinical characteristics of the two populations with different immune status and illustrated differentiated risk factors for death in them.

## INTRODUCTION

In December 2019, a cluster of patients with pneumonia of unknown cause was linked to a seafood wholesale market in Wuhan, China [1]. Shortly after that, a previously unknown betacoronavirus was discovered through unbiased sequencing in samples from patients with such pneumonia [2]. The novel β-coronavirus was named SARS-CoV-2 by International Committee on Taxonomy of Viruses (ICTV) and WHO had officially named this new disease as corona virus disease 2019 (COVID-19) [3]. Since then, the COVID-19-confirmed cases increased rapidly. As of August 28, 2020, a total of 24257989 confirmed cases and 827246 related deaths have been reported worldwide [4]. Moreover, COVID-19 cases have been reported in more than 200 countries, areas or territories. On January 30, 2020, WHO announced that the event constituted a Public Health Emergency of International Concern (PHEIC), indicating that a big threat to global health has been posed by the novel coronavirus infections [5].

The novel coronavirus, namely SARS-CoV-2, belongs to the betacoronavirus family, as SARS-CoV and MERS-CoV do. However, SARS-CoV-2 is relatively unique. It is less deadly but has a relatively covert characteristic compared to the other two coronaviruses [3]. SARS-CoV-2 could be transmitted from person to person and the basic reproductive number (R0) of it is estimated to be 1.4-3.9, which is higher than that of MERS-CoV (0.50-0.92) and nearly the same as SARS-CoV (2.3-3.7) [3, 6]. Moreover, unlike SARS-CoV, asymptomatic patients infected with SARS-CoV-2 could be a source of transmission, which jeopardizes the screening of infected people by temperature measurements or by overt signs and symptoms [3]. There is now no special treatment or vaccine for this disease and the only treatment is supportive, which might be a contribution to the high death rate in the elderly patients. The overall death rate of COVID-19 is 2.3% but elderly patients are more likely to be seriously ill [7]. The fatality rates of patients over 60 are higher than the average, and the deaths of them accounts for over 50% of the total deaths nationwide in China [8, 9]. Furthermore, the immune status of healthy people ≥ 70 is different from those < 70 [10]. Thus, the clinical courses and risk factors of patients aged 70 or above and those under 70 are likely to differ considerably. In this study, we investigated the different clinical characteristics between patients ≥ 70 and those < 70. The study aims to provide useful information for exploring differentiated prognostic factors in COVID-19 patients.

## RESULTS

### Demographics and clinical characteristics

There were 222 patients in total. At the end of the follow-up, 41 patients were dead, the rest 181 were still alive. Patients were divided into an over-70 group with ages ≥ 70 and an under-70 group with ages < 70, as presented in Table 1. The median age of all patients was 51.5 (interquartile range, IQR 34.0-65.3). The minimum age of these patients was 21 and the maximum age of them was 97. The median age for the over-70 group patients was 77.0 (IQR 73.0-82.0) and that for the under-70 group was 45.0 (33.0-56.0). Meanwhile, 90 patients were male, accounting for a percentage of 40.54% among all involved patients. Common comorbidities of these patients included chronic cardiovascular disease (44 cases, 19.82%), chronic pulmonary disease (19 cases, 8.56%), chronic liver disease (10 cases, 4.5%), chronic kidney disease (7 cases, 3.15%) and diabetes (24 cases, 10.81%). 26 patients (11.71%) had a smoking history. Among all symptoms, fever was the most common one (157 cases, 70.72%), followed by dry cough (90 cases, 40.54%), fatigue (51 cases, 22.97%), expectoration (48 cases, 21.62%), pharyngalgia (26 cases, 11.71%) and so on. As for the treatment, antibacterial therapy was applied in 138 patients (62.16%), antiviral therapy was administered in 124 patients (55.86%), glucocorticoids were used in 55 patients (24.77%) and immunoglobulin was employed in 47 patients (21.17%).

**Table 1:** Baseline characteristics of patients with SARS-CoV-2 infection.

**Characteristics**	**All patients (n=222)**	**Age ≥70 (n=37)**	**Age<70 (n=185)**	***P* value^a^**
Ages				
Age, median (IQR)	51.5 (34.0-65.3)	77.0 (73.0-82.0)	45.0 (33.0-56.0)	P<0.0001
Minimum age	21	70	21	
Maximum age	97	97	69	
Gender		0.14
Male	90(40.54)	19(51.35)	71(38.38)	
Female	132(59.46)	18(48.65)	114(61.62)	
Chronic medical illness			
Chronic cardiovascular disease	44(19.82)	19(51.35)	25(13.51)	<0.001
Chronic pulmonary disease	19(8.56)	5(13.51)	14(7.57)	0.39
Chronic liver disease	10(4.5)	2(5.41)	8(4.32)	0.88
Chronic kidney disease	7(3.15)	3(8.11)	4(2.16)	0.17
Diabetes	24(10.81)	8(21.62)	16(8.65)	0.04
Smoking	26(11.71)	6(16.22)	20(10.81)	0.51
Symptoms			
Fever	157(70.72)	27(72.97)	130(70.27)	0.74
Dry cough	90(40.54)	14(37.84)	76(41.08)	0.71
Expectoration	48(21.62)	12(32.43)	36(19.46)	0.08
Pharyngalgia	26(11.71)	6(16.22)	20(10.61)	0.51
Dyspnoea	32(14.41)	15(40.54)	17(9.19)	<0.001
Diarrhea	19(8.56)	3(8.11)	16(8.65)	0.83
Headache	17(7.66)	2(5.41)	15(8.11)	0.82
Muscle ache	25(11.26)	2(5.41)	23(12.43)	0.34
Fatigue	51(22.97)	6(16.22)	45(24.32)	0.28
Chest pain	7(3.15)	2(5.41)	5(2.7)	0.33
Loss of appetite	16(7.21)	4(10.81)	12(6.49)	0.56
Treatment			
Antibacterial agents	138(62.16)	25(67.57)	113(61.08)	0.46
Antiviral agents	124(55.86)	19(51.35)	105(56.76)	0.55
Glucocorticoids	55(24.77)	10(27.03)	45(24.32)	0.73
Immunoglobulin	47(21.17)	8(21.62)	39(21.08)	0.94

Abbreviations: IQR, interquartile range.

^a^ P values indicate differences between over-70 and under-70 group patients. P < 0.05 was considered statistically significant.

Compared to patients under 70 years old (n=37, 16.67%), those who were 70 or older (n=185, 83.3%) were more likely to have chronic cardiovascular disease (P<0.001) and diabetes (P=0.04,). Also, those over-70 patients were more inclined to develop dyspnoea (P<0.001). Otherwise, there were no differences in the occurrence rates of the aforementioned symptoms or in the prevalences of chronic medical illnesses between the two groups of patients. No differences of treatment were observed between the two groups, either.

### Laboratory parameters in COVID-19 patients

There were plenty of differences in laboratory findings between under-70 patients and over-70 ones (Table 2). For blood routine, higher white blood cell count (P<0.001), neutrophil count (P<0.001) and lower lymphocyte count (P<0.001), platelet count (P=0.002) were observed in 70 or older patients compared with those under 70. The level of C-reactive protein (CRP) was tremendously higher in over-70 patients (P<0.001), indicating a more serious infection. In patients who are 70 or older, higher levels of aspartate aminotransferase (AST; P<0.001), urea (P<0.001), creatinine (Cr; P=0.006), lactate dehydrogenase (LDH; P<0.001), creatine kinase (CK; P<0.001) were evidenced. Also, weaker immune functions were more common in these patients as evidenced by lower CD3, CD4, CD8, CD19 (all P<0.001) counts and lower CD3 (P<0.001), CD4 (P<0.001), CD8 (P=0.02), CD16+56 (P<0.001) percents in them. As for antibody and complement levels, patients in the over-70 group had a significantly lower level of IgM (P=0.01) and significantly higher levels of IgG (P=0.004), IgA (P=0.003), C4 (P=0.002) compared to those in the other group.

**Table 2:** Laboratory findings in the two groups.

	**Normal range**	**Total(n=222)**	**Age≥70 (n=37)**	**Age<70 (n=185)**	***P* value^a^**
**Blood routine**					
White blood cell count, ×10^9^ /L	3.5-9.5	4.84(3.99-6.22)	5.56(4.32-8.56)	4.76(3.97-5.87)	<0.001
Neutrophil count, ×10^9^ /L	1.8-6.3	3.01(2.15-4.29)	4.17(3.04-7.4)	2.89(2.05-4.04)	<0.001
Lymphocyte count, ×10^9^ /L	1.1-3.2	1.12(0.78-1.58)	0.76(0.51-0.97)	1.24(0.86-1.65)	<0.001
Platelet count, ×10^9^ /L	125-350	189(151-239)	168(121-218)	195(162-246)	0.002
C-reactive protein, mg/L	0-5	12(1-57.3)	71.4(30.8-144)	7.09(0.4-34.6)	<0.001
**Liver function**	
Alanine aminotransferase, U/L	9-50	19(14-31)	20(14.5-29)	19(13-31)	0.84
Aspartate aminotransferase, U/L	15-40	25(19-34.3)	33(26.5-55.5)	23(19-31)	<0.001
**Kidney function**	
Urea, mmol/L	3.1-8.0	4.21(3.36-5.55)	7.1(5.25-12.3)	4(3.16-5.16)	<0.001
Creatinine, μmol/L	57-97	56(48-73)	88(56.5-135)	53(47-67.5)	0.006
**Myocardial function**	
Lactate dehydrogenase, U/L	120-250	214(171-294)	336(247-496)	206(168-265)	<0.001
Creatine kinase, U/L	50-310	63(39-122)	128(72.5-226)	57(37-99)	<0.001
**Immune function**	
CD3(%)	56-86	68(57.4-75.9)	52.7(43.3-62.3)	70.1(60.1-76.3)	<0.001
CD3 count, /μL	723-2737	702(393-1095)	283(174-426)	776(534-1132)	<0.001
CD4(%)	33-58	39.1(30.4-45)	28.3(21.5-37.3)	40.3(33.9-45.5)	<0.001
CD4 count, /μL	404-1612	396(239-670)	143(92-245)	453(302-697)	<0.001
CD8(%)	13-39	23.7(19-29.5)	19.1(11.9-26.8)	24.2(19.9-30.5)	0.02
CD8 count, /μL	220-1129	257(135-405)	103(51.5-166)	278(181-426)	<0.001
CD4/CD8	ratio 0.9-2.0	1.61(1.21-2.19)	1.41(0.96-2.58)	1.64(1.25-2.13)	0.09
CD19(%)	5-22	13.7(9.88-20.2)	13.6(7.66-22.3)	13.7(9.95-20.1)	0.59
CD19 count, /μL	80-616	144(95.5-209)	73(42-141)	156(110-219)	<0.001
CD16+56 (%)	5-26	13.7(8.15-21.4)	22.7(14.4-40)	12.4(7.52-18.4)	<0.001
CD16+56 count, /μL	84-724	125(81-202)	123(77-207)	126(83-203)	0.97
**Complement level**	
IgG, g/L	8-16	12.7(10.1-16.5)	15.9(12.3-18.5)	12.2(9.93-15.9)	0.004
IgM, g/L	0.4~3.45	1.09(0.85-1.43)	0.94(0.66-1.28)	1.11(0.86-1.48)	0.01
IgA, g/L	0.76~3.9	1.94(1.47-2.58)	2.46(1.68-3.32)	1.87(1.46-2.45)	0.003
IgE, IU/mL	<100	29.6(17.3-102)	32.9(17.8-96.4)	28.8(17.3-105)	0.93
C3, g/L	0.81-1.6	0.85(0.74-0.98)	0.85(0.72-0.96)	0.85(0.74-0.98)	0.35
C4, g/L	0.1-0.4	0.24(0.18-0.31)	0.29(0.19-0.38)	0.24(0.18-0.31)	0.002

^a^P values indicate differences between over-70 and under-70 group patients. P < 0.05 was considered statistically significant.

### Severity of disease and complications

The spectrum of diseases and complications for each patient were recorded in Table 3. The median time interval from symptom onset to admission was 7 (IQR 4-11.25) days. There was no significant difference between the under-70 group and over-70 group (P=0.25). Hospital stay median was 15.5 (IQR 11-19) days and the number of death cases was 41 (18.47%) in total. Patients under 70 had a significantly shorter hospital stay (15 days, IQR 11-19) and a significantly lower death rate (19 cases, 10.27%) than their over-70 counterparts (18 days, IQR 14-22.5, P=0.02; 22 cases, 59.46%, P<0.001).

**Table 3:** Baseline spectrum of disease and complications.

**Characteristics**	**All patients (n=222)**	**Age ≥70 (n=37)**	**Age<70 (n=185)**	***P* value^a^**
Duration from onset of symptoms to admission, median (IQR), days	7(4-11.25)	7(3-10)	7(4-12)	0.25
Hospital stay, median (IQR), days	15.5(11-19)	18(14-22.5)	15(11-19)	0.02
Death	41(18.47)	22(59.46)	19(10.27)	<0.001
Disease severity		<0.001
Mild	28(12.61)	0	28(15.14)	
Moderate	70(31.53)	2(5.41)	68(36.76)	
Severe	31(13.96)	10(27.03)	21(11.35)	
Critical	93(41.89)	25(67.57)	68(36.76)	
Complication	
ARDS	54(24.32)	31(83.78)	23(12.43)	<0.001
Acute liver dysfunction	43(19.37)	12(32.43)	31(16.76)	0.03
Acute kidney injury	29(13.06)	15(40.54)	14(7.57)	<0.001
Bacterial infection	12(5.41)	6(16.22)	6(3.24)	0.005

Abbreviations: IQR, interquartile range; ARDS, acute respiratory distress syndrome.

^a^ P values indicate differences between over-70 and under-70 group patients. P < 0.05 was considered statistically significant.

On admission, the degree of severity of COVID-19 was categorized as mild in 28 patients (12.61%), moderate in 70 patients (31.53%), severe in 31 patients (13.96%) and critical in 93 patients (41.89%). The disease severities of the 2 groups of patients were different (P<0.001). The percentages of patients with mild and moderate disease in under-70 patients were higher than those in the over-70 group. Correspondingly, severe and critical cases accounted for a lower proportion in patients in the under-70 group than those in the other group. During hospitalization, many of the patients received a diagnosis of acute respiratory distress syndrome (ARDS) (54 cases, 24.32%), followed by acute liver dysfunction (ALD) (43 cases, 19.37%), acute kidney injury (AKI) (29 cases, 13.06%) and bacterial infection (12 cases, 5.41%). Patients in the over-70 group had significantly higher incidences of all the above-mentioned complications than those who are under 70 (all P<0.05, Table 3).

### Factors related to fatality in the over-70 group patients

**Figure 1:**
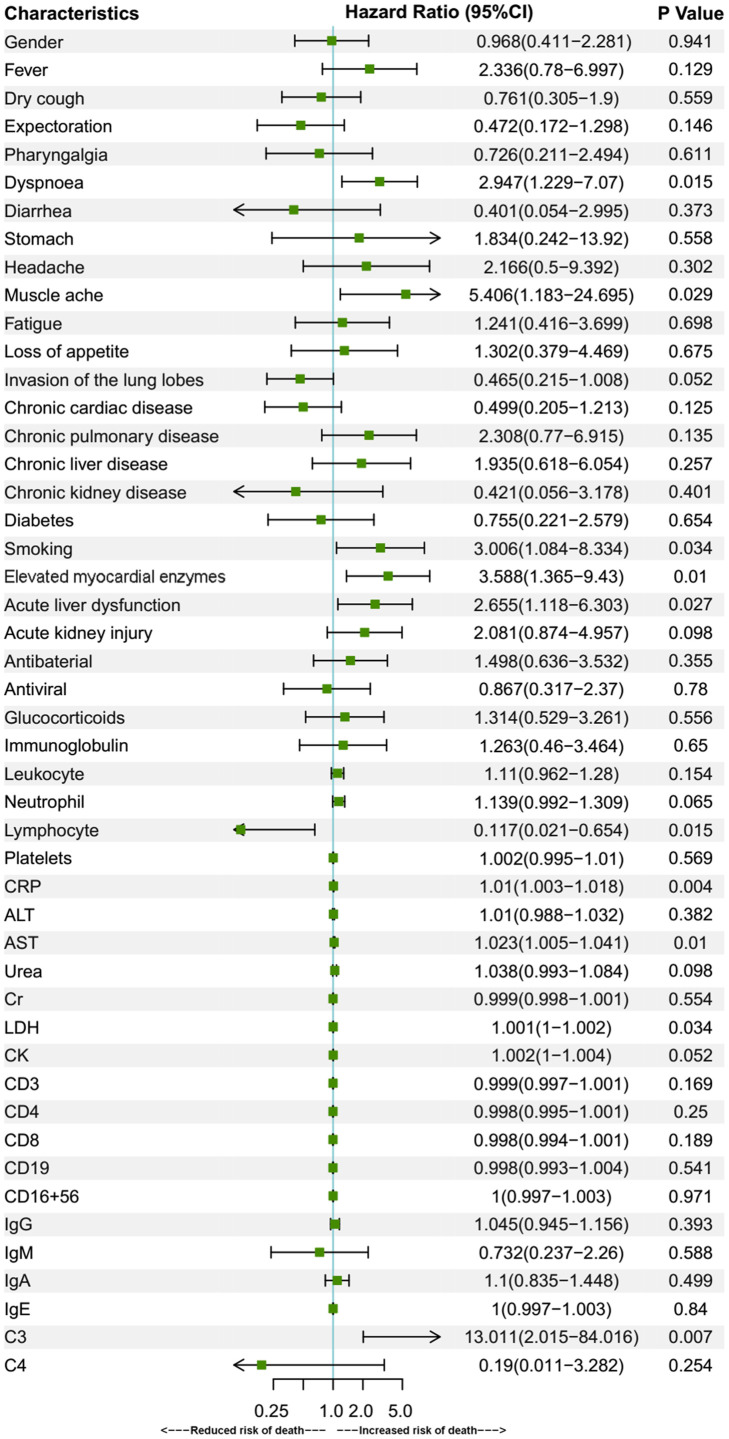
**Univariate Cox regression for prognostic factors of over-70 group patients.** Univariate Cox regression analysis of fatality risk factors in over-70 patients. Elevated myocardial enzymes were defined if serum LDH or CK was above the upper reference limit. Data are represented as means with 95% confidence intervals. Abbreviations: CI, confidence interval; CRP, C-reactive protein; ALT, alanine aminotransferase; AST, aspartate aminotransferase; Cr, creatinine; LDH, lactate dehydrogenase; CK, creatine kinase.

**Figure 2:**
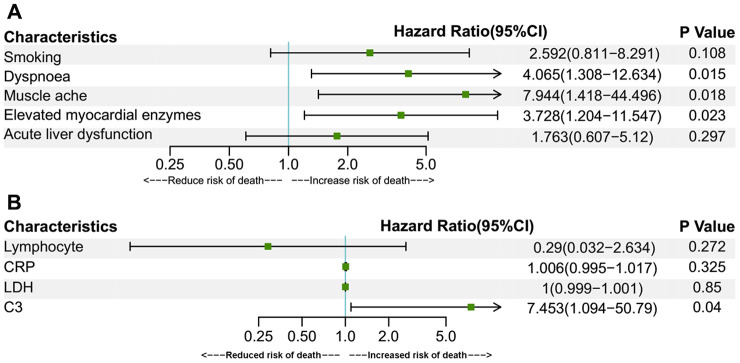
**Multivariate Cox regression for prognostic factors of over-70 group patients.** Multivariate Cox regressions were performed for fatality risk factors of symptoms, chronic medical illness, complications, elevated myocardial enzymes (**A**) and other laboratory findings (**B**) identified in the univariate Cox regression analysis. Elevated myocardial enzymes were defined if serum LDH or CK was above the upper reference limit. Data are represented as means with 95% confidence intervals. Abbreviations: CI, confidence interval; CRP, C-reactive protein; LDH, lactate dehydrogenase.

To analyze the risk factors for fatal outcomes in 70-year-old or older patients, univariate Cox regression was applied in the over-70 group (Figure 1). The hazard ratio (HR) and 95% confidence interval (CI) were shown in the columns right to the chart. Gender was shown to have no effect on the likelihood of death in over-70 patients (P=0.941). Symptoms of dyspnoea (HR 2.947, CI 1.229-7.07, P = 0.015), muscle ache (HR 5.406, CI 1.183-24.695, P = 0.029), a history of smoking (HR 3.006, CI 1.084-8.334, P=0.034), elevated myocardial enzymes (HR 3.588, CI 1.365-9.43, P = 0.01), complication of ALD (HR 2.655, CI 1.118-6.303, P = 0.027), elevated laboratory parameters of CRP (HR 1.01, CI 1.003-1.018, P=0.004), AST (HR 1.023, CI 1.005-1.041, P=0.01), LDH (HR 1.001, CI 1-1.002, P=0.034) and C3 (HR 13.011, CI: 2.015 – 84.016, P=0.007) were all correlated with an increased incidence of fatal outcomes. Meanwhile, an increased parameter of lymphocyte (HR 0.117, CI 0.021–0.654, P=0.015) was associated with a decreased risk of fatality. The models used in the univariate analysis were tested by likelihood ratio test, Wald test and Score (log-rank) test. All the three methods supported the significance of the models. Then multivariate Cox regression analyses for the above prognostic factors were performed (Figure 2). In multivariate analyses, only syndromes of dyspnoea (HR 4.065, CI 1.308-12.634, P = 0.015), muscle ache (HR 7.944, CI 1.418–44.496, P = 0.018), elevated myocardial enzymes (HR 3.728, CI 1.204-11.547, P = 0.023), and increased C3 (HR 7.453, CI 1.094-50.79, P = 0.04) remained to be correlated with an increased death risk.

### Factors related to fatality in the under-70 group patients

**Figure 3:**
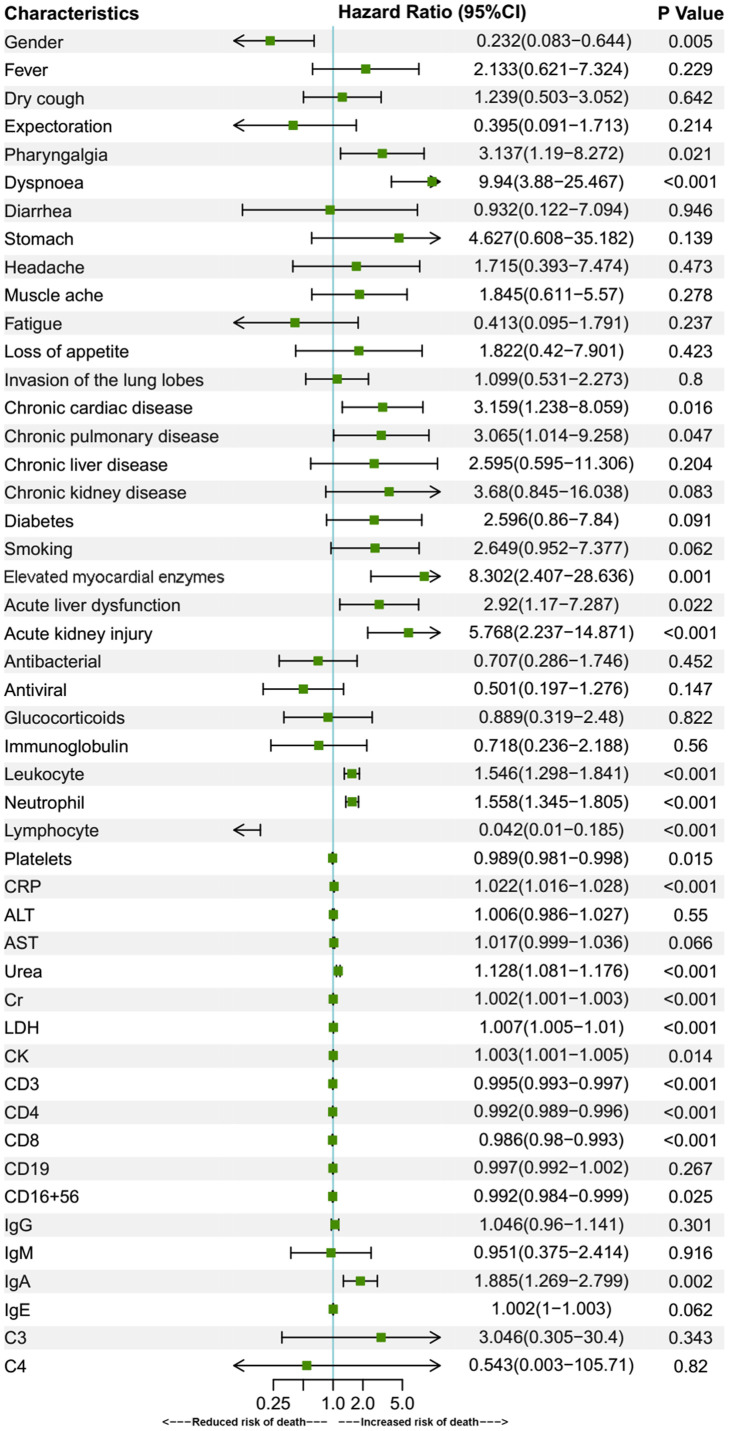
**Univariate Cox regression for prognostic factors of under-70 group patients.** Univariate Cox regression analysis of fatality risk factors in under-70 patients. Elevated myocardial enzymes were defined if serum LDH or CK was above the upper reference limit. Data are represented as means with 95% confidence intervals. Abbreviations: CI, confidence interval; CRP, C-reactive protein; ALT, alanine aminotransferase; AST, aspartate aminotransferase; Cr, creatinine; LDH, lactate dehydrogenase; CK, creatine kinase.

**Figure 4:**
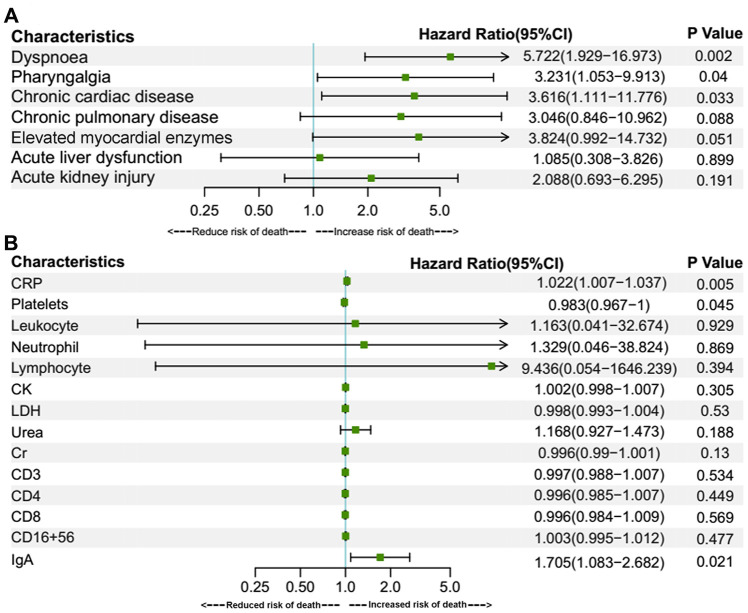
**Multivariate Cox regression for prognostic factors of under-70 group patients.** Multivariate Cox regressions were performed for fatality risk factors of symptoms, chronic medical illness, complications, elevated myocardial enzymes (**A**) and other laboratory findings (**B**) identified in the univariate Cox regression analysis. Elevated myocardial enzymes were defined if serum LDH or CK was above the upper reference limit. Data are represented as mean with a 95% confidence interval. Abbreviations: CI, confidence interval; CRP, C-reactive protein; CK, creatine kinase; LDH, lactate dehydrogenase; Cr, creatinine.

Univariate Cox regression was also applied in the under-70 group (Figure 3). Contrary to the over-70 group, gender has a significant effect on fatality in this group and the female gender is correlated with a decreased risk of death (HR 0.232, CI 0.083–0.644, P = 0.005). Otherwise, symptoms of pharyngalgia (HR 3.137, CI 1.19–8.272, P = 0.021), dyspnoea (HR 9.94, CI 3.88–25.467, P < 0.001), comorbidities of chronic cardiac disease (HR 3.159, CI 1.238-8.059, P = 0.016), chronic pulmonary disease (HR 3.065, CI 1.014-9.258, P = 0.047), elevated myocardial enzymes (HR 8.302, CI 2.407–28.636, P = 0.001), complications of ALD (HR 2.92, CI 1.17-7.287, P = 0.022), AKI (HR 5.768, CI 2.237-14.871, P < 0.001), elevated laboratory parameters of leukocyte (HR 1.546, CI 1.298-1.841, P < 0.001), neutrophil (HR 1.558, CI 1.345-1.805, P < 0.001), CRP (HR 1.022, CI 1.016-1.028, P<0.001), urea (HR 1.128, CI 1.081-1.176, P<0.001), Cr (HR 1.002, CI 1.001-1.003, P<0.001), LDH (HR 1.007, CI 1.005-1.01, P<0.001), CK (HR 1.003, CI 1.001-1.005, P=0.014), IgA (HR 1.885, CI 1.269–2.799, P = 0.002) were all related with an increased fatality rate. Meanwhile, increased lymphocyte (0.042, 0.01–0.185, P < 0.001), platelets (HR 0.989, CI 0.981-0.998, P=0.015), CD3 (HR 0.995, CI 0.993-0.997, P<0.001), CD4 (HR 0.992, CI 0.989-0.996, P<0.001), CD8 (HR 0.986, CI 0.98-0.993, P<0.001), CD16+56 (HR 0.992, CI 0.984-0.999, P=0.025) were associated with a decreased risk of fatality. Subsequently, multivariate Cox regression analyses for the above prognostic factors were performed (Figure 4). In multivariate analyses, only dyspnoea (HR 5.722, CI 1.929–16.973, P = 0.002), pharyngalgia (HR 3.231, CI 1.053–9.913, P = 0.04), chronic cardiac disease (HR 3.616, CI 1.111-11.776, P = 0.033), increased CRP (HR 1.022, CI 1.007-1.037, P=0.005), IgA (HR 1.705, CI 1.083–2.682, P = 0.021) and decreased platelets (HR 0.983, CI 0.967-1, P=0.045) remained to be associated with an increased death risk.

### Clinical outcomes

**Figure 5:**
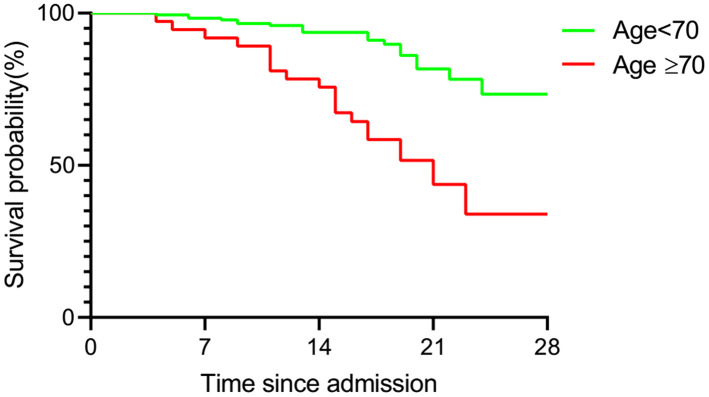
**Kaplan-Meier analysis of over-70 and under-70 group patients.** Statistical analysis of the correlation of age with survival of COVID-19 patients. Survival curve showed a poorer prognosis in over-70 group patients.

To determine the prognoses of COVID-19 patients in both groups, the survival probabilities of both age groups were analyzed by Kaplan-Meier analysis (Figure 5). The survival analysis revealed that patients with an age equal or greater than 70 had a lower cumulative survival rate than those younger than 70 (HR 6.412, CI 2.739-15.01, P<0.001).

## DISCUSSION

In the present study, we described the clinical characteristics as well as the risk factors related to the fatality of COVID-19 patients. The patients were divided into an over-70 group and an under-70 group. SARS-CoV-2 infection caused a higher mortality rate in the first group than the latter one. Of the 37 over-70 COVID-19 patients included, more than 90% were severe or critical and the mortality rate of them is 59.46%. In contrast, a less-than-50% percentage of severe or critical cases and a death rate of 10.27% were observed in the under-70 patients. A longer hospital stay was also observed in the aged group patients with a median time of 18 days, compared to that of 15 days in the younger group patients. Several factors were noticed to be relevant to a higher fatality rate of the over-70 group patients, such as dyspnea, muscle ache and increased C3. In comparison, the factors for the younger group patients included dyspnoea, pharyngalgia, chronic cardiac disease, increased CRP, IgA and decreased platelets.

The most common symptoms of COVID-19 patients in this research were fever, dry cough, fatigue and expectoration, which were generally consistent with those in other studies [9, 11, 12]. Patients also had underlying diseases such as chronic heart disease, chronic pulmonary disease, chronic liver disease and chronic kidney disease. Among them, chronic heart disease and chronic pulmonary disease are the most common comorbidities. In the research by Guan et al., the prevalences of coronary heart disease and chronic obstructive pulmonary disease (COPD) were 2.5% and 1.1%, respectively [9]. Meanwhile, the prevalences of cardiovascular disease and COPD by Wang et al. were 14.5% and 2.9% [11]. In comparison, the prevalences are high in ours, numbering 19.82% and 8.56%, respectively. This might contribute to a large proportion of severe-to-critical patients and a high death rate in our study. The prevalences of other comorbidities in these studies are similar and might contribute less to disease severity and fatality. An important complication of COVID-19 is acute myocardial injury (AMI), which is reflected specifically by troponin I and creatine kinase-MB. The total incidences of AMI are 21%, 7.2% and 12% in the research by Wang L et al. (21%) [8], Wang et al. (7.2%) [11] and Huang et al. (12%) [12]. However, due to the lack of specific biomarkers in our study, the incidence of AMI was not available. In addition, the incidence rate of ARDS was also higher in the over-70 group patients compared to the under-70 ones, which might be correlated with a higher occurrence rate of dyspnoea in that group. Significant differences were also observed in the incidence of complications such as ALD, AKI, and bacterial infection. The occurrence rates of them were higher in patients in the over-70 group. Similarly, the occurrence rates of ARDS, ALD and AKI are all higher than those reported by Guan et al. [9]. This might be correlated with higher prevalences of chronic pulmonary disease, chronic kidney disease and chronic liver disease in our study. It may also contribute to high proportions of severe-to-critical COVID-19 patients in our research, especially for those in the over-70 group.

The prominent features of the over-70 patients in our study were distinctly increased severity and death rate. The reported percentage of severe-to-critical patients in the whole population was 18.5% and the death rate was 2.3% [7], which are much lower than those of 55.85% and 18.47% in the present study. In addition, in the study by Chen et al. involving 99 patients [13], the mean ages of the patients were 55.5 in contrast to an median age of 51.5 in our study. The proportions of the patients with an age ≥ 70 in the study of Chen et al. and ours were similar (15% vs. 16.7%), but the total death rates differ (11% vs. 18.47%) [13]. The differences might be explained by a high percent of severe-to-critical patients in our research (55.85%) compared to that in the whole population (18.5%) or to the percent of patients admitted to ICU in the study of Chen et al. (23%). In the study by Guan et al. including 1099 patients [9], the total death rate was 1.4% and the death rate of severe cases was 8.1%. The low death rate in their study may also be attributed to a much lower proportion of severe cases (15.7%) compared to ours. The data of most of the patients in the above-mentioned studies was collected in January. Thus, the characteristics of these patients were comparable. The above differences might be partially accounted for by the different classification standards in these studies. However, the alterations in classification standards doesn’t seem decisive in explaining these differences. The high death rate of the patients and high proportion of severe-to-critical patients in our study might be better explained by the fact that Renmin Hospital of Wuhan University was a designated hospital for severe COVID-19 patients.

In addition to case severity and death rate, the immune status of the two groups of COVID-19 patients in our study also differs, as revealed by the significantly lower counts of CD3, CD4, CD8, CD19 and percentages of CD3, CD4, CD8, CD16+56 in the aged group compared to the other group. This is consistent with the results of our previous research that indicated the lower levels of CD3^+^ T cell, CD4^+^ T cell, CD8^+^ T cell, B cell (CD19^+^) and NK cell (CD16^+^ 56^+^) in severe COVID-19 cases and their correlations with the course of those cases [14]. Consistently, lymphocyte count was also lower in the over-70 group than in the under-70 group. In previous research, lymphocyte count was reduced in patients admitted to ICU compared to non-ICU patients [11, 12]. It could be partly explained by the fact that lymphocytes could be killed by coronaviruses due to the damage of the cytoplasmic components or the activation of apoptosis [15, 16]. Lymphocyte counts are also indicators of the immune system. In a previous study investigating reference values for T lymphocytes in healthy people, the numbers of CD4^+^ and CD8^+^ lymphocytes differed significantly between people aged 70 or older and those younger participants [10]. Considering the different counts of lymphocytes and their subpopulations between the two age groups in our study, the immune status are likely to differ significantly between them. In addition to lymphocytes, the levels of AST, urea, Cr, LDH, and CK are also higher in the aged group patients in our study. The increased levels of them accorded with a higher incidence rate of corresponding organ injury and higher prevalences of organ comorbidities in COVID-19 patients with ages ≥ 70.

Older age is a known risk factor for death in COVID-19 patients [8, 17, 18]. This is reflected by a higher death rate and a lower cumulative survival rate in the over-70 group in our study. In addition to age, there were several risk factors for death, as revealed by Cox regression analyses in the present study. In the over-70 COVID-19 patients, the factors included dyspnoea, muscle ache, elevated myocardial enzymes and increased C3. In the under-70 patients, the factors involved chronic cardiac disease, dyspnoea and increased IgA. Among the factors mentioned above, myocardial comorbidity is a predictor of death, as was reported in previous research [8, 17]. This might be attributed to the distribution of angiotensin-converting enzyme 2 (ACE2), the key host cellular receptor of SARS-CoV-2, in cardiac cells. Previous research has shown that patients with underlying heart disease exhibited an increased ACE2 expression; if these people were infected by the virus, they might be exposed to a high risk of heart attack and a critically ill condition [19]. Besides, respiratory comorbidities and complications were also reported to be risk factors in previous research [8, 18]. Dyspnoea was probably a manifestation for impaired lung function and might be related to ARDS [8]. These respiratory manifestations may lead to respiratory failure and fatality. An increased C3 level might be a sign of an activated complement system, which regulates a systemic inflammatory response and contributes to the pathologic outcomes of coronavirus infection [20]. Increased IgA may be a sign of massive virus infection since SARS-CoV-2 behaves like other respiratory viruses and yield the production of protective secretory IgA in infected individuals [21]. Thus, these indexes reflected the death risks in the two age groups of COVID-19 patients with different immune status.

There are some limitations in this study. First of all, the proportion of severe and critical patients as well as the fatality rate were obviously higher than the officially disclosed. This might be due to the fact that Renmin Hospital of Wuhan University was a designated hospital for severe COVID-19 patients. Secondly, the number of the elderly patients included in this study was lower than that of the younger patients, which might lead to some random errors. In addition, this is an retrospective observational study and is not able to lead to causal conclusions.

In conclusion, COVID-19 patients aged 70 or older were more likely to develop dyspnoea, have lower levels of lymphocytes, neutrophils, platelets, and were more prone to have organ comorbidities and corresponding complications than younger patients. The percentage of severe cases among the aged group patients was also higher, correlating with a higher death rate among them. The risk factors for death included dyspnoea, muscle ache, elevated myocardial enzymes, elevated C3 in over-70 patients and dyspnoea, pharyngalgia, chronic cardiac disease, increased CRP, IgA, decreased platelets in under-70 patients. Our research compared the clinical characteristics of the two populations with different immune status and provided differentiated risk factors for death in them.

## MATERIALS AND METHODS

### Data sources

The study was in compliance with the edicts of the Declaration of Helsinki and was approved by the Ethics Committee of Renmin Hospital of Wuhan University (No. WDRY2020-K009). Written informed consent was waived by the same committee in light of the quick-spreading epidemic around the world and the evaluation that anonymous data here involved no potential risk to patients and no link between the patients and the researchers. The patients who were confirmed as SARS-CoV-2 infection by nucleic acid detection and chest CT between January 13, 2020 and February 4, 2020 in Renmin Hospital of Wuhan University, had intact laboratory test results and were still alive 24 h after hospitalization were included in this study. The last day of follow-up was February 27, when the follow-up time of all survival patients reached 28 days. The primary end point was survival and death until February 27, 2020 and the living status of those patients was confirmed at that time. The clinical data involved information such as age, gender, comorbidities, symptoms, treatment, laboratory results, hospital stay, clinical outcomes, spectrum of underlying diseases and complications. Patients were assigned into two groups according to their ages: an over-70 group with patients’ ages ≥ 70, and an under-70 group with patients’ ages < 70.

### Data collection

The diagnosis of COVID-19 was established if SARS-CoV-2 nucleic acid was detected by quantitative real-time PCR in respiratory or blood specimens and the patient has radiological manifestations of pneumonia. The criteria were based on the Interim Guidance for Novel Coronavirus Pneumonia (5^th^ edition) issued by National Health Commission of the People’s Republic of China [22]. Patients of COVID-19 were divided into 4 types according to the guidance. The classifications of COVID-19 were listed as follows: mild (mild clinical symptoms, without pneumonic manifestations on chest imaging), moderate (fever and respiratory clinical symptoms, with pneumonic manifestations on chest imaging), severe (in accordance with any of the following: dyspnoea, respiratory frequency ≥ 30/minute; blood oxygen saturation ≤ 93% during rest; PaO2/FiO2 ratio ≤ 300mmHg) and critical (in accordance with any of the following: respiratory failure requiring mechanical ventilation; shock; complicating other organ failure that requires intensive care).

The development of complications was confirmed by three physicians according to the following criteria: ARDS was defined with reference to the Berlin definition [23]; ALD was defined if serum alanine aminotransferase (ALT) or AST was above the upper reference limit; AKI was identified with reference to Clinical Practice Guidelines on AKI [24]; bacterial infection was defined with blood white blood cell count > 9.5×10^9^ /L.

### Statistical analysis

Continuous variables were expressed as mean, median and interquartile range values, as appropriate. Categorical variables were summarized as counts and percentages. Means or medians for continuous variables were compared using independent group t tests when the data were normally distributed; otherwise, the Mann-Whitney test was used. Proportions for categorical variables were compared using the χ^2^ test with or without Yate’s correlation, or the Fisher’s exact test, depending on the situations. All the analyses were performed with GraphPad Prism 8. The clinical, laboratory variables, comorbidities and complications were included in univariate Cox analyses using R language v3.6.1 with packages “survival” and “survminer”. The risk factors identified in the univariate analyses were subsequently analyzed by multivariate Cox regression analyses. For all parameters, the cutoff of significance was set at P < 0.05.
